# A Novel Remote Patient and Medication Monitoring Solution to Improve Adherence and Persistence With Inflammatory Bowel Disease Therapy (ASSIST Study): Protocol for a Randomized Controlled Trial

**DOI:** 10.2196/40382

**Published:** 2022-12-15

**Authors:** Jordan Axelrad, Millie Long, Sara Horst, Anita Afzali, Tamar Sapir, Kristina Fajardo, Kara De Felice, Robert Sandler, Raymond Cross

**Affiliations:** 1 Division of Gastroenterology Department of Medicine NYU Grossman School of Medicine New York, NY United States; 2 Division of Gastroenterology and Hepatology Department of Medicine University of North Carolina School of Medicine Chapel Hill, NC United States; 3 Division of Gastroenterology, Hepatology, and Nutrition Department of Medicine Vanderbilt University School of Medicine Nashville, TN United States; 4 Division of Gastroenterology Department of Medicine University of Cincinnati College of Medicine Cincinnati, OH United States; 5 Synchronyx Boca Raton, FL United States; 6 Division of Gastroenterology Department of Medicine University of Maryland School of Medicine Baltimore, MD United States

**Keywords:** remote therapy monitoring, connected health, patient engagement, Crohn disease, ulcerative colitis, inflammatory bowel disease, mobile phone

## Abstract

**Background:**

Inflammatory bowel diseases (IBDs) are chronic inflammatory conditions of the gastrointestinal tract. Although adherence to IBD therapies is associated with improved clinical outcomes, overall adherence is poor. Consequently, there is a critical need to develop interventions that monitor adherence in real time and identify reasons for nonadherence to support clinical teams in initiating effective interventions. Recently, electronic- and web-based platforms have been developed to monitor adherence and guide interventions. A novel remote therapy monitoring (RTM) technology, the Tappt digital health system, has been developed to monitor real-time medication adherence patterns through smart label technologies, capture patient-reported outcomes and barriers to care, and process patient data through algorithms that trigger personalized digital and human touch points between clinical visits. Such a digital health solution enables care teams to proactively identify and mitigate nonadherence and worsening clinical outcomes.

**Objective:**

We propose a 12-month multicenter randomized controlled trial to assess the effectiveness of the Tappt digital health system on adherence, clinical outcomes, and health care use among patients diagnosed with IBD starting a new oral or subcutaneous therapy.

**Methods:**

The digital health system intervention will provide automatic measurement of medication adherence via smart labels for pill bottles or injectors as well as a monitoring platform for providers. The system will prompt patients to complete a two-item assessment of symptoms monthly using the PRO-2 scales for UC and Crohn disease, from which increased symptoms will be alerted to providers. Participants will be randomized 2:1 to the intervention group or the control group, which will receive standard of care. All participants are required to complete questionnaires at baseline as well as at 12, 26, and 52 weeks. Assuming an adherence rate of 0.65 and 0.9 among control and intervention participants, respectively, we will need to enroll 123 participants: 82 (66.7%) in the intervention group and 41 (33.3%) controls. We will compare adherence as measured by the medication possession ratio, defined as the number of days of supply of medication obtained during the observation period out of the total number of days in the observation period, in participants using the RTM versus those receiving standard of care. We will also compare clinical outcomes and health care use in participants using the RTM versus those receiving standard of care.

**Results:**

We anticipate starting recruitment in December 2022.

**Conclusions:**

Effective medication adherence monitoring and intervention programs need to be cost-efficient, pose little or no burden to the patient, record reliable data in real time, and provide actionable insights to the health care team. We anticipate the Tappt digital health system to improve the medication possession ratio, clinical outcomes, and health care use compared with standard of care.

**Trial Registration:**

ClinicalTrials.gov NCT05316584; https://clinicaltrials.gov/ct2/show/NCT05316584

**International Registered Report Identifier (IRRID):**

PRR1-10.2196/40382

## Introduction

### Background

Inflammatory bowel diseases (IBDs), comprising Crohn disease (CD) and ulcerative colitis (UC), are chronic, progressive, inflammatory conditions of the gastrointestinal tract that affect up to 3 million Americans [1]. IBD is thought to be driven by inappropriate immune responses to an altered gut microbiome, with a disease course characterized by remitting and relapsing episodes of inflammation, or flares.

Most patients with a chronic condition require long-term therapy. Evidence has demonstrated that suboptimal adherence is associated with poor clinical outcomes, including increased IBD activity and associated complications, worsened quality of life (QoL) [2], and higher health care use and costs [3]. In addition, nonadherence to biologic therapies may increase the risk for developing antidrug antibodies, leading to loss of response to therapy. Despite this, adherence to IBD therapies remains suboptimal. A recent survey administered to 322 patients with IBD found that 55% reported nonadherence, including forgetting or skipping medication owing to not feeling well [4]. Overall adherence to IBD medications ranges from 40% to 80%, depending on the patient population, medication class, data sources, and adherence quantification measures [5].

Adherence to oral IBD medications such as aminosalicylates and thiopurines ranges from 50% to 93% [6]. Studies evaluating adherence to biologic therapies are conflicting. Using a medication possession ratio (MPR)—calculated as the number of days of supply of medication obtained during the observation period out of the total number days in the observation period—of <100% as a definition of nonadherence in 365 patients with IBD treated with biologic therapies, adherence was highest for intravenous formulations (70%-83%) compared with subcutaneous biologics (47%-50%) [7]. Conversely, in a large multicenter prospective cohort study where adherence was defined as ≥80% using a visual analog scale, the majority of patients with CD and UC were considered adherent (88% and 86%, respectively) [8]. However, when comparing across medication classes, adherence was lowest for patients on rectal (68%) and oral (83%) therapies and highest for intramuscular (88%) and parenteral (87%) therapies [8]. Similar results were found in a recent study of 112 patients with IBD in the Manitoba cohort: 81% of the patients were considered adherent to their therapy using an MPR cutoff value of ≥90% [9].

The variance in adherence is in part due to inconsistent measurement. Objective and validated approaches include pill counts, refill counts, and pharmacological or biochemical markers; subjective approaches include validated measures, visual analog scales, and patient interviews. Some approaches may work better in certain settings, given that they measure adherence in different ways and at different time points [10]; for example, although validated measures are effective in clinical trials, they can be burdensome and time consuming in real-world practice. Furthermore, they do not capture day-to-day variability in adherence because the adherence measure summarizes medication-taking behaviors over a designated period [3]. Similarly, pharmacy records and insurance databases provide data that can be used to calculate MPR and percentage of days covered by the prescriptions, but they do not provide information on medication intake patterns and do not capture use of samples [10,11]. Patient interviews are the most efficient way to gather a complete picture of patient adherence, but they can be unreliable, given that patients tend to overestimate their adherence [10].

In recent years, electronic- and virtual-based platforms have been developed to monitor adherence routinely and guide appropriate interventions. These remote therapy monitoring (RTM) systems include electronic pill caps, electronic pill and needle boxes, bidirectional SMS text message reminders, and patient-facing mobile apps [10,12-14]. Although some of these have been successful at monitoring and improving adherence, they are limited by their battery requirement and cost as well as the survey fatigue of respondents. Furthermore, these solutions and apps collect data inconsistently and are not synchronized with clinical teams’ workflows in a real-time manner to identify patients at risk for nonadherence and drive actionable follow-up.

Synchronyx, which is based in Boca Raton, Florida, United States, has developed a scalable RTM and patient engagement solution called the Tappt digital health system. It consists of smart battery-free labels affixed to medication packages that transmit encrypted adherence data from the patient’s smartphone through a secure server; a patient-facing web application with access to personalized data and educational resources; and a centralized cloud-based and password-protected clinician portal that summarizes patients’ medication intake behaviors in real time at individual and aggregate levels, collects patients’ barriers to care and patient-reported outcomes (PROs), and triggers alerts to designated clinical teams.

### Preliminary Work

#### IBD Home Automated Telemanagement

For patients with IBD, we previously developed the IBD Home Automated Telemanagement (HAT) system, which consists of a laptop computer, an electronic weighing scale, and a physician web portal. The laptop computer and scale made up the patient home unit. The laptop computer administered questions on patient symptoms, adherence, and medication side effects and delivered educational messages, whereas the scale was used to regularly measure body weight [15,16].

We performed a 6-month open-label trial to assess the feasibility and patient acceptance of the IBD HAT system in patients with IBD. We predetermined that ≥80% adherence with self-testing would demonstrate feasibility. Patient acceptance of the HAT system was evaluated using the IBD HAT attitudinal survey. In total, 27 patients with IBD were recruited from the University of Maryland and Veterans Affairs Hospital in Baltimore, Maryland, United States. Participants continued to receive standard IBD care in addition to the weekly HAT sessions.

Of the 27 participants, 25 (93%) successfully completed the 6-month study. During the study, 89% (22/25) of the participants were adherent with weekly self-testing, and 91% (23/25) said that self-testing was not complicated. Of the 25 participants, 18 (73%) reported that they felt safer using the system, and 23 (91%) stated that they would agree to use the self-testing program in the future. Self-reported adherence with IBD medications was 90% throughout the study. Clinical disease activity, disease-specific QoL, and patient knowledge improved compared with baseline [17].

We then pursued a 12-month randomized clinical trial to assess the effectiveness of the UC HAT system compared with best available care (BAC). We modified the symptom diary and alert criteria to make them specific to UC and added action plans and electronic messaging for participants to communicate with the research team. Adults with UC from the University of Maryland and Veterans Affairs Hospital in Baltimore were randomized to self-testing with the UC HAT system weekly or BAC. All participants underwent visits at baseline and every 4 months. Disease activity was measured using the Seo index [18,19], disease-specific QoL was measured using the Inflammatory Bowel Disease Questionnaire [20], and medication adherence was measured using a medication adherence scale [21]. We randomized 47 patients, and 29 (62%) completed the study. After adjustment for baseline QoL scores, participants in the UC HAT arm had a strong trend toward decreased Seo index scores from baseline (mean –11.9, SD 6.6; *P*=.08). Furthermore, after adjustment for baseline disease knowledge, participants in the UC HAT arm had a significant increase in QoL scores from baseline (mean 12.5, SD 5.9; *P*=.04), whereas participants in the BAC arm had nonsignificant decreases in QoL scores from baseline (mean –3.8, SD 5.3; *P*=.47). The difference in change in QoL scores from baseline between the UC HAT and BAC arms at 12 months was significant. Self-reported adherence to medical therapy was low in both groups at baseline (BAC: 45% and UC HAT: 40%). Adherence rates increased in both groups but were not significantly different at any time point after baseline. Both arms had poor IBD knowledge at baseline, with improvement at the 12-month follow-up; however, there was not a significant difference between the UC HAT and BAC arms at the 12-month follow-up [22]. Our results suggested that the randomization process was inadequate because participants in the UC HAT arm had higher rates of immune suppression use and greater impairments in QoL than participants in the BAC arm. We suspect that the baseline differences in favor of the BAC arm impaired our ability to demonstrate a treatment effect of the UC HAT system. This hypothesis is supported by the findings of clinically important improvements in disease activity and QoL from the baseline to the 12-month visit in the UC HAT arm. Furthermore, after adjustment for baseline disease knowledge, a significant improvement in QoL was noted in the UC HAT participants.

#### Telemedicine for Patients With IBD

We then conducted a multicenter randomized controlled trial to assess the effectiveness of telemedicine for patients with IBD (TELE-IBD) weekly (TELE-IBD W) or every other week (TELE-IBD EOW) compared with BAC. Patients completed self-assessments via SMS text messages, assessment of weight was completed through use of an electronic scale and manually entered by the participant, and educational messages were sent either weekly or twice weekly throughout the trial. We recruited 348 participants (n=116, 33.3%, TELE-IBD W; n=115, 33%, TELE-IBD EOW; and n=117, 33.6%, BAC controls), including 236 (67.8%) participants with CD and 112 (32.2%) with UC or indeterminate colitis. Of the 348 participants, 259 (74.4%) completed the final 12-month visit. Clinical indices of CD activity using the Harvey-Bradshaw Index (HBI) decreased in the control, TELE-IBD EOW, and TELE-IBD W groups (*P*=.16). The decreases in HBI scores over time were significant in all groups (*P*<.001) but not different among the groups (*P*=.18). Clinical indices of UC activity using the Simple Clinical Colitis Activity Index (SCCAI) decreased in the control, TELE-IBD EOW, and TELE-IBD W groups (*P*=.41), but they were significant only in the controls (*P*=.01) and not different among the groups (*P*=.25). IBD QoL scores increased in the control, TELE-IBD EOW, and TELE-IBD W groups (*P*=.42) but were significant only in the TELE-IBD EOW group (*P*=.03) and not different among the groups (*P*=.95). However, use of health care resources was different among the groups, with participants in the TELE-IBD arms experiencing lower total and IBD-related hospitalization rates and higher number of noninvasive diagnostic tests, telephone calls, and electronic messages [23]. In addition, those in the TELE-IBD arms had a greater improvement in Crohn’s and Colitis Knowledge scores [24] than those in the standard of care group. General self-efficacy scores improved in all arms during the trial but were not different among the groups [25,26]. In an analysis of intervention participants only, adherence increased with depressive symptoms in those who were aged ≤40 years (*P*=.02), but there was no association between depressive symptoms and adherence in those who were aged >40 years (*P*=.53) [27]. A follow-up qualitative study demonstrated that participants identified clear benefits of the TELE-IBD system, including a better understanding of the disease process, monitoring of symptoms, and feeling of connection to the health care team. Both adherent and nonadherent participants preferred a flexible system that was personalized, included targeted education, and promoted self-management [28].

### Aims

On the basis of the aforementioned preliminary work demonstrating that technology has the capacity to improve medication adherence and clinical outcomes, there is a critical need to integrate interventions that are affordable and practical to monitor adherence in real time. In addition, interventions that aim to identify reasons for nonadherence, intentional or unintentional, should be prioritized to support clinical teams in tailoring adherence interventions to each unique patient [10,29]. As such, we propose a 12-month multicenter randomized controlled trial to assess the effectiveness of an RTM solution, the Tappt digital health system, on adherence, clinical outcomes, and health care use among patients with IBD starting a new oral or subcutaneous therapy. Specifically, we will (aim 1) compare adherence as measured by the MPR in participants using the digital health system versus those receiving standard of care and (aim 2) compare clinical outcomes and health care use in participants using the digital health system versus those receiving standard of care.

## Methods

### Overview and Population

The proposed study is a multicenter randomized controlled trial to be conducted over 12 months. Participants in the intervention arm will be encouraged to verify medication adherence using the Tappt digital health system. This system will provide automatic measurement of medication adherence via smart labels for pill bottles or injectors as well as a monitoring platform for providers. In addition, the Tappt system will prompt patients to complete an assessment of symptoms monthly using the PRO scale, 2-item version (PRO-2) for UC and CD; if symptoms worsen, alerts will be triggered to providers. Participants randomized to the control group will receive standard of care. All participants are required to complete questionnaires at baseline and at 12, 26, and 52 weeks.

### Study Schedule

All participants are required to complete electronic data entry forms at baseline and at 12, 26, and 52 weeks. Adherence, disease activity, QoL, PROs, IBD self-efficacy, and health care use will be measured at each time point during the 1-year study (Table 1). Demographic and clinical information will be collected at the baseline visit.

**Table 1:** Schedule of events for the study.

Instrument	Category	Source	Baseline	Week 12	Week 26	Week 52
Demographics	Demographics	Patient	✓			
Clinical history	Clinical information	Provider or site	✓			
Medication adherence	Adherence	Patient		✓	✓	✓
MARS-5^a^	Adherence	Patient	✓	✓	✓	✓
Health care use	Health care use	Patient	✓	✓	✓	✓
Modified Harvey-Bradshaw Index	Disease activity: CD^b^	Patient	✓	✓	✓	✓
Simple Clinical Colitis Activity Index	Disease activity: UC^c^	Patient	✓	✓	✓	✓
C-reactive protein	Disease activity	Provider or site	✓	✓	✓	✓
Calprotectin	Disease activity	Provider or site	✓	✓	✓	✓
Simple endoscopic score: CD	Disease activity	Provider or site	✓	✓	✓	✓
Mayo Endoscopic Score	Disease activity	Provider or site	✓	✓	✓	✓
PROMIS^d^ Global Health Scale	Quality of life	Patient	✓	✓	✓	✓
PROMIS anxiety	Mental health	Patient	✓	✓	✓	✓
PROMIS depression	Mental health	Patient	✓	✓	✓	✓
PROMIS sleep disturbance	Mental health	Patient	✓	✓	✓	✓
PROMIS fatigue	Disease activity	Patient	✓	✓	✓	✓
PROMIS pain interference	Disease activity	Patient	✓	✓	✓	✓
PROMIS physical function	Frailty	Patient	✓	✓	✓	✓
IBD^e^ self-efficacy	Self-efficacy	Patient	✓	✓	✓	✓

^a^MARS-5: Medication Adherence Report Scale-5.

^b^CD: Crohn disease.

^c^UC: ulcerative colitis.

^d^PROMIS: Patient-Reported Outcome Measurement Information System.

^e^IBD: inflammatory bowel disease.

### Study Population

Patients will be recruited for this study from 5 clinical centers at the University of Maryland School of Medicine, University of Cincinnati School of Medicine, NYU Grossman School of Medicine, University of North Carolina School of Medicine, and Vanderbilt University School of Medicine.

Inclusion criteria will include (1) aged ≥18 years; (2) documented IBD based on usual diagnostic criteria, including clinical symptoms and findings from endoscopy, radiology studies, and histology [30]; (3) initiation of treatment with a new oral or subcutaneous treatment for IBD; (4) access to a smartphone with a reliable data plan or Wi-Fi access; and (5) ability to understand the protocol and provide informed consent in English. Exclusion criteria will include (1) inability to speak and read English or comply with the study protocol; (2) the presence of an ileostomy, colostomy, ileoanal pouch anastomosis, or ileorectal anastomosis; (3) initiation of oral corticosteroids only without concurrent use of an oral or subcutaneous maintenance therapy; (4) imminent surgery within the next 60 days; (5) history of short bowel syndrome; or (6) uncontrolled medical or psychiatric disease.

### Study Conduct

Eligible patients will be approached by research staff at the time of routine clinical visits or via telephone and provide informed consent. All of the participants’ concomitant medications will be continued during the study. Corticosteroids will be tapered at the discretion of the investigator. Addition of new IBD medications will be allowed during the study. If the new medication is an eligible oral or subcutaneous addition to existing therapy, this will be tracked using the approach that was used to track the original medication during the initial treatment. If the new medication replaces the initial therapy, the replacement medication will be similarly tracked.

Participants will be randomized 2:1 to receive either remote monitoring or standard of care using a random permuted block design with randomly varied block sizes. Participants will be stratified based on study site and disease subtype (CD vs UC or indeterminate colitis). We chose to stratify on these variables because they could affect the response to the intervention; stratification will ensure balance of these covariates among the study groups. The randomization order will be computer generated, and group assignment will be made when a new participant is enrolled into the study by the clinical site. The site investigators and research staff will remain blinded to the randomization order. The participants and physicians will not be blinded to the group assignment; however, we will attempt to minimize measurement bias by collecting outcome data through electronic data capture directly from participants.

### Intervention Group

Eligible patients will complete a baseline survey gathering demographic and clinical information. Upon enrollment, the intervention participants will activate their account to access the patient-facing Tappt web application, which includes the individual participants’ medication regimen information, upcoming doses, and adherence data. The web application also includes a curated resource library with links to educational material on the Crohn’s and Colitis Foundation website, expert-led video modules on each IBD medication class, and training videos on how to use the system and seek support. The web application also allows patients to respond to surveys related to nonadherence or PROs. The system uses algorithms to send participants personalized alerts and reminders for medication doses or survey completion through SMS text messages.

After providing training to the intervention participants on how to use the Tappt system, the research team will input into the system the intervention participants’ deidentified information as well as the medication to be tracked, dose, and frequency of dosing. The research team will then assign smart labels to the participants’ medication regimen, which will be used by the participants to record their medication use. The research team will also walk each study participant through the Tappt system and explain how to label and scan medication and how to complete all required surveys. Along with the first medication, the corresponding smart labels to be affixed to the pill bottle, pen, or syringe container of the newly prescribed medication will be shipped to the participants. Participants will receive sufficient labels for dosing of 90 days of the medication to be tracked, plus an additional 2 to 6 labels to account for lost or damaged labels and dose escalation. Participants will receive enough labels until their next assessment (ie, a participant on mesalamine 2.4 g daily will receive 90 labels). Changes in dose of medications for the intervention participants will be monitored on a weekly basis by study coordinators. Any change in dosing will be updated on the Tappt clinician portal; labels will be reassigned to the new dosing frequency, and a new set of smart labels will be sent to the participants for the next 90-day dosing period.

At the time of taking a medication dose, participants will scan the label with their mobile phone to verify that they are taking the medication. Upon scanning, participants will immediately receive a notification on their device indicating that the label was successfully scanned and that their medication adherence was updated in their profile. Each day that a medication is due to be taken, participants will receive a reminder through SMS text message notifying them of their medication schedule for the day. If participants fail to scan the label at a given time (as determined by their specific medication regimen), they will receive an SMS text message at the end of the day with a link to respond to a survey that captures reasons for nonadherence.

Participants will also complete a PRO-2 assessment at baseline and then monthly for the entire 12 months of the study triggered by the Tappt system through an HTML link sent to patients by SMS text message [31,32]. For participants with CD, the PRO-2 assessment includes abdominal pain and liquid stool frequency. For participants with UC or indeterminate colitis, the PRO-2 assessment includes rectal bleeding and stool frequency. The research team will also be able to send the PRO-2 survey to patients at any given time, at their discretion, if patients are experiencing a flare or at the time of a change in medication dose. Items for the PRO-2 assessment vary based on disease type (refer to the Outcomes and Outcome Measures section).

**Figure 1:**
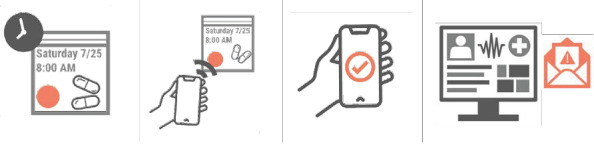
Study intervention: the Tappt remote therapy monitoring digital health system.

Clinical teams can review their participants’ data in real time through the secure cloud-based provider-facing Tappt portal. To reduce monitoring burden among clinical teams, the system’s algorithms prompt automated email alerts to the clinical team when participants’ adherence or PROs fall below predetermined thresholds, when a participant’s regimen is approaching refill date, or when a participant reports an error on their profile. These alerts can support clinical teams in following up with patients in a timely manner to reduce risk for poor outcomes (Figure 1).

For oral medications, mean adherence <80% in a 2-week period will trigger an alert. For subcutaneous medications, 2 missed doses of adalimumab and 1 missed dose of other biologics will trigger an alert. If alerts are triggered for nonadherence, a clinical nurse, pharmacist, or social worker will contact the participant to identify barriers to adherence; remediation will be initiated, if possible. For participants with CD, a PRO-2 abdominal pain score of 2 (moderate) or higher or a liquid stool frequency score of >4 will trigger an alert. For participants with UC or IBD type undetermined, a rectal bleeding score of 2 or a stool frequency score of 2 will trigger an alert (obvious blood most of the time or blood alone passes and stool frequency >2 stools more than normal, respectively). A clinical nurse will contact the participant if a symptom-based alert is triggered.

### Control Group

The standard of care for participants in this study is modeled after the standard of care at all study sites. Standard of care is based on current evidence-based guidelines, including a comprehensive assessment, a guideline-concordant therapy plan, scheduled and as-needed clinic visits, scheduled and as-needed telephone calls, and administration of educational fact sheets about disease-specific topics when appropriate. Personnel used to provide standard of care at each site will vary and may include nurse coordinators, advanced practice providers, social workers, psychologists, dietitians, pharmacists, and other ancillary staff.

### Outcomes and Outcome Measures

#### Overview

The outcome measures outlined in the following sections will be assessed at baseline and at 12, 26, and 52 weeks after initiating treatment. All outcome measures will be captured electronically and will not require a study or office visit.

The primary outcome of the proposed study will be the difference in mean MPR between the intervention and control groups during the 12-month study. The mean MPR will be calculated as follows:

MPR = number of days’ supply of medication obtained during observation period / total number of days in observation period.

If a participant discontinues therapy but initiates treatment with another eligible medication, the MPR will continue to be calculated. Any delay in initiating new treatment will be adjusted for the total number of days in the observation period (ie, if drug A is discontinued and drug B is started 2 weeks later, the observation period will be 365 – 14 = 351 days); for example, if a participant is prescribed oral mesalamine (1.2 g), 4 tablets daily, for 90 days (dispense 360) with 2 refills between months 6 and 12, the MPR would be 100%. Likewise, if a participant is prescribed ustekinumab 90 mg subcutaneous every 8 weeks (dispense 1 syringe) with 2 refills between months 6 and 12, the MPR would be 66%. Data on medication use will be self-reported by participants at each time point. However, we will randomly validate participant report via chart review of pharmacy data for 20% of the participants. Participants will report the date the medications of interest were filled as well as the number of days’ supply of medications filled. Self-reported adherence will be assessed with the Medication Adherence Report Scale-5 questionnaire (Table 2). Scores for each item are summed to give a total score, with higher scores indicating higher levels of reported adherence [33].

The secondary outcome of the proposed study will be the difference in health care use between the intervention and control groups during the 12-month study. For health care use, we will use a composite end point, including counts of hospitalizations, urgent care or emergency room visits, IBD-related surgeries, telephone calls (primary care and IBD provider), endoscopic procedures, imaging examinations, and blood and stool testing. We will also assess new prescriptions for corticosteroids (prednisone and budesonide) at each time point during the study period. The total events will be adjusted per 100 patient years of follow-up because the 2 groups will be unequal in number. In addition, we will evaluate each outcome individually. The relatedness of hospitalization and urgent care or emergency room visits to IBD will be determined as well. Information will be obtained from patient self-report at each assessment. This approach has been validated in patients with IBD using a modification of the Canadian Community Health Survey [34]. However, we will validate participant self-report via chart review for 20% of the participants, randomly selected.

**Table 2:** Medication Adherence Report Scale-5 questionnaire.

Item	Scoring				
	Always	Often	Sometimes	Rarely	Never
I forget to take my medication	1	2	3	4	5
I alter my medication dose	1	2	3	4	5
I stop taking my medication for a while	1	2	3	4	5
I decide to miss out a dose of my medication	1	2	3	4	5
I take less medication than instructed	1	2	3	4	5

#### Assessment of Disease Activity

To measure disease activity in participants with CD, we will assess a modified HBI without including the item on the presence of an abdominal mass (Table 3). HBI scores of <5, 5 to 7, 8 to 16, and >16 correlate with symptomatic remission, mildly active symptoms, moderately active symptoms, and severely active symptoms, respectively. A decrease in the HBI score of 3 or 4 correlates well with a symptomatic response [35].

In addition to calculating clinical remission and response using the aforementioned measures, we will create an outcome of steroid-free remission or response based on whether the patient was on concurrent steroids at the time of assessment. In patients with UC and indeterminate colitis, disease activity will be assessed with the SCCAI (Table 4). An SCCAI score of <3 has been shown to correlate with symptomatic remission, whereas a decrease of 2 points has been shown to correlate with a symptomatic response [36].

**Table 3:** Modified Harvey-Bradshaw Index.

Item	Scoring
General well-being	Well: 0, fair: 1, poor: 2, terrible: 3
Abdominal pain	None: 0, mild: 1, moderate: 2, severe: 3
Number of liquid stools per day	Each liquid stool: 1
Arthralgia	Yes: 1, no: 0
Uveitis	Yes: 1, no: 0
Erythema nodosum	Yes: 1, no: 0
Aphthous ulcers	Yes: 1, no: 0
Pyoderma gangrenosum	Yes: 1, no: 0
Anal fissure	Yes: 1, no: 0
New fistula	Yes: 1, no: 0
Abscess	Yes: 1, no: 0

**Table 4:** Simple Clinical Colitis Activity Index.

Item	Scoring				
	0	1	2	3	4
Bowel frequency (day)	1 to 3	4 to 6	7 to 9	>9	N/A^a^
Bowel frequency (night)	None	1 to 3	4 to 6	N/A	N/A
Urgency of defecation	None	Hurry	Immediately	Incontinence	N/A
Blood in stool	None	Trace	Occasionally frank	Usually frank	N/A
General well-being	Very well	Slightly below par	Poor	Very poor	Terrible
Aphthous ulcers	None	Present	N/A	N/A	N/A
Uveitis	None	Present	N/A	N/A	N/A
Erythema nodosum	None	Present	N/A	N/A	N/A
Arthralgia	None	Present	N/A	N/A	N/A

^a^N/A: not applicable.

We will calculate steroid-free remission as described in the previous paragraph. To assess disease activity using the Tappt system, we will use the PRO-2 assessments for CD [31] and UC [32]. These measures of PROs have been validated in IBD and demonstrate an association between improvements in patient-reported symptoms and clinical remission and response [37,38]. For CD, the PRO-2 assessment comprises questions regarding the frequency of bowel movements in the prior week and the degree of abdominal pain (Table 5). PRO-2 scores of 8, 14, and 34 correlated with Crohn’s Disease Activity Index [39] scores of 150, 220, and 450, respectively. A Crohn’s Disease Activity Index score of <150 is consistent with clinical remission, whereas a score of >220 is consistent with moderate to severe disease. In lieu of calculating a total score, an average liquid stool frequency per day of >4 or an average abdominal pain score of 2 (moderate) or higher will trigger an alert.

For UC and indeterminate colitis, a PRO-2 assessment specific for UC will be used. It comprises questions related to stool frequency and rectal bleeding (Table 6). A score of ≥2 for stool frequency or rectal bleeding will trigger an alert.

**Table 5:** Patient-reported outcome scale, 2-item version, for patients with Crohn disease.

Variable	Day								7-day average		Weighting factor		Total
	1	2	3	4	5	6	7						
Number of liquid or very soft stools	—^a^	—	—	—	—	—	—	—		× 2 = —		—	
Abdominal pain (0=none, 1=mild, 2=moderate, 3=severe)	—	—	—	—	—	—	—	—		× 5 = —		—	

^a^To be filled in by health care staff.

**Table 6:** Patient-reported outcome scale, 2-item version, for patients with ulcerative colitis.

Stool frequency	Rectal bleeding	Score
Normal number of stools	No blood seen	0
1 to 2 stools more than normal	Streaks of blood with stool less than half the time	1
3 to 4 stools more than normal	Obvious blood with stool most of the time	2
≥5 stools more than normal	Blood alone passes	3

In addition, the results of quantitative C-reactive protein and fecal calprotectin tests, if available, will be extracted from the medical record at each time point. Research staff will use the value closest to the time of assessment within 30 days. C-reactive protein correlates well with endoscopic disease activity (*r*=0.56), and values <5 mg/dL have negative predictive values for endoscopic disease activity ranging from 29% to 61% [40-42]. Likewise, fecal calprotectin correlates well with endoscopic disease activity (*r*=0.53) but has better negative predictive values for endoscopic disease activity ranging from 71% to 97% using a value of <250 mcg/g [42,43]. Similarly, we will collect information on endoscopic disease activity, if available. For UC and IBD type undetermined, we will use the Mayo Endoscopic Score (MES; Table S1 in Multimedia Appendix 1). The MES assessment was modified to remove friability in the mild disease category. An MES of 0 to 1 is consistent with endoscopic remission [44].

For patients with CD, we will assess endoscopic disease activity with the Simple Endoscopic Score (Table S2 in Multimedia Appendix 1). A score of <3 is consistent with inactive disease, 3 to 6 mildly active, 7 to 15 moderately active, and ≥16 severely active [45,46]. We will also record new steroid use (oral or topical treatment) in the interval between assessments.

#### Assessment of QoL and PRO Measures

The Patient-Reported Outcome Measurement Information System is a National Institutes of Health–funded instrument that assesses patients’ self-reported health over a 7-day period [47]. Patients report global health as well as different components of physical, mental, and social health, including physical function, anxiety, depression, fatigue, sleep disturbance, and pain interference; higher scores indicate poorer health (Tables S3-S9 in Multimedia Appendix 1). Patient-Reported Outcome Measurement Information System scores are standardized to the general population where a T-score of 50 is the standardized normal, with an SD of 10. Therefore, these measures can be considered within normal limits (<55), mild (55 to <60), moderate (60 to <70), and severe (≥70) based on domain scores measured in the general population in the United States. Minimally important differences of 2 to 6 points have been reported for other disease states, including chronic pain, stroke, osteoarthritis, and cancer [48].

#### Self-efficacy

Self-efficacy is a perception of a patient’s ability to engage in the skills necessary to master a new challenge when facing obstacles. An IBD-specific self-efficacy scale has been developed (Table S10 in Multimedia Appendix 1). The 29-item instrument includes questions on managing medical care, stress and emotions, and symptoms and disease, as well as maintaining remission. Responses range from 1=not sure at all to 10=totally sure. Scores range from 29 to 290, with higher scores correlating with greater self-efficacy [49].

#### Health Care Use

Health care use will be measured using the Canadian Community Health Survey (Table S11 in Multimedia Appendix 1).

### Data Management

Outcome measures from participants will be collected via direct data capture through use of electronic data entry forms sent to participants via email. Information on health care use will be collected by research staff at each site and recorded in the source documents. The data will then be entered electronically into electronic case report forms. All data will be maintained, archived, retrieved, and distributed by a computer system.

### Statistical Analyses

Baseline analyses will include tabulations of demographic and clinical variables of the study participants. Chi-square tests and 2-tailed *t* tests will be used to compare these groups to determine whether baseline differences exist. The distribution of each continuous variable will be inspected for outliers to determine whether a parametric or nonparametric approach should be applied. The Wilcoxon rank sum test will be used for any variable that does not meet the statistical assumptions of the *t* test. In addition, analyses will be conducted stratified by clinical site to determine whether the sites are comparable across the groups of interest. If differences are detected, these variables will be evaluated as potential confounders in the regression models.

For study aim 1, we will compare the mean MPR ratio between the intervention and standard of care groups using *t* tests. For study aim 2, we will compare differences in health care use between the groups using the Wilcoxon ranked sum test because we expect that the data will not be normally distributed.

For the secondary aims of clinical outcomes and health care use, dichotomized outcomes will be compared in a similar fashion with bivariate analyses. For health care use, the outcome will be investigated in quartiles of health care use, and specific outcomes of interest selected a priori will be investigated (eg, need for hospitalization for IBD, need for IBD surgery, and initiation of corticosteroids).

All analyses will be completed using intention-to-treat principles. Assuming 2 intervention participants for every 1 control, with adherence rates of 0.65 among controls and 0.9 in intervention participants and with a type I error rate of 0.05 and power of 0.9, we will need to enroll 123 participants: 82 (67%) in the intervention group and 41 (33%) controls. We may enroll more patients pending our initial recruitment and data capture.

Although we will make every effort to obtain complete data from all randomized participants, we anticipate that there could be as much as 20% loss to follow-up by 12 months. To detect potential bias by analysis of only the collected data, we plan first to compare patients with data with those without data at a given time point on baseline characteristics to determine characteristics of those who do not provide complete data. We will also perform sensitivity testing to see whether estimates of intervention effects differ if only patients with complete data are analyzed. Depending upon the extent of missingness, we will also consider imputation methods that use multiple regression predictions for imputation.

### Ethics Approval

We will seek approval for this study from the institutional review boards of each participating institution.

## Results

We anticipate starting recruitment in December 2022.

## Discussion

### Study Expectations

The proposed study addresses 2 high-priority research areas in the field of IBD. First, it will assess a novel RTM digital health technology to improve adherence and clinical outcomes. Second, it will determine whether enhanced remote monitoring improves clinical outcomes compared with standard of care. As CD and UC are chronic illnesses that require continual medical management to prevent disease relapse and complications and as nonadherence has been linked to poor clinical outcomes and greater health care use, interventions that improve adherence are greatly needed. If our RTM digital health system is effective at improving adherence as we anticipate, we expect superior clinical outcomes and decreased use of unnecessary health care use such as unplanned clinic visits, steroid prescriptions, emergency room visits, and hospitalizations. In addition, if a system is patient-centric, easy to use, and can interface with existing clinical workflows so that clinical team members can intervene in a timely fashion for patients at risk for poor outcomes, it will improve the care experience and lives of patients living with IBD.

The results of this study can lay the foundation for a larger study to input data from the Tappt digital health system into the electronic medical record so that all members of the health care team can easily view the results. A larger study would also facilitate inclusion of communities with more diverse study populations and populations with health disparities. In addition to advancing the science on remote monitoring in chronic illnesses and methods to improve adherence, the remote monitoring system could be accessed by patients not only to track adherence and symptoms but also as a link to vetted educational resources and their personalized data. Furthermore, the system might be useful to large integrated health systems and provider networks or to payers to improve adherence, reduce health care use, and contain costs. In addition, given the recent reimbursement expansion for RTM under Medicare, provider teams can benefit from generating recurring revenue and improve the patient care experience derived from RTM digital health systems.

### Few Difficulties or Limitations Anticipated

We anticipate few difficulties or limitations in conducting the proposed study. Enrollment of 123 participants over 12 months among 5 sites (approximately 2 per site per month) is a very reasonable goal as each site is a large referral center for the care of patients with IBD. In addition, we are not restricting enrollment to patients initiating treatment with a biologic or small molecule, which will increase the pool of eligible patients. If recruitment is challenging, we can recruit additional sites to participate. We have included sites in the Northeast, mid-Atlantic, Midwest, and South, which should increase the diversity of the patient population studied. However, because IBD is underrepresented in populations consisting of people of color, our participants will not be as diverse as the general population at each site. Moreover, a digital platform may not benefit disadvantaged people with limited access to technology or technological literacy. Finally, although the COVID-19 pandemic seems to be easing, it is possible that new variants of the virus will emerge, resulting in restricted access for in-person visits. We do not anticipate that prescribing patterns will change if this occurs, and we have implemented remote consent and data collection to mitigate these barriers to enrollment and capture of outcome data.

## Appendix

Multimedia Appendix 1 Supplemental tables.

## Abbreviations

BAC: best available care
CD: Crohn disease
HAT: Home Automated Telemanagement
HBI: Harvey-Bradshaw Index
IBD: inflammatory bowel disease
MES: Mayo Endoscopic Score
MPR: medication possession ratio
PRO: patient-reported outcome
PRO-2: patient-reported outcome scale, 2-item version
QoL: quality of life
RTM: remote therapy monitoring
SCCAI: Simple Clinical Colitis Activity Index
TELE-IBD EOW: telemedicine for patients with inflammatory bowel disease every other week
TELE-IBD W: telemedicine for patients with inflammatory bowel disease weekly
TELE-IBD: telemedicine for patients with inflammatory bowel disease
UC: ulcerative colitis
