# Supplementary material for: A Novel Remote Patient and Medication Monitoring Solution to Improve Adherence and Persistence With Inflammatory Bowel Disease Therapy (ASSIST Study): Protocol for a Randomized Controlled Trial
Source: JMIR Res Protoc. 2022 Dec 15;11(12):e40382. doi: 10.2196/40382 (PMC9801266; doi:10.2196/40382)
Supplement: Multimedia Appendix 1 [file resprot_v11i12e40382_app1.docx]

**Supplemental Table 1: Mayo Endoscopic Score for UC**

| Findings of flexible sigmoidoscopy/colonoscopy | Disease Activity | Score |
| --- | --- | --- |
| Normal | Inactive disease | 0 |
| Erythema, decreased vascular pattern | Mild | 1 |
| Marked erythema, absent vascular pattern, friability, erosions | Moderate | 2 |
| Spontaneous bleeding, ulcerations | Severe | 3 |

**Supplemental Table 2: Simple Endoscopic Score for Patients with Crohn’s Disease**

|  | Ileum | Right colon | Transverse colon | Rectum | Total |
| --- | --- | --- | --- | --- | --- |
| Presence and size of ulcers (0=none, 1=less than 0.5 cm, 2=0.5-2.0 cm, 3>2.0 cm |  |  |  |  |  |
| Ulcerated surface (0=none, 1=<10%, 2=10-30%, 3=>30%) |  |  |  |  |  |
| Affected surface (0=unaffected, 1=<50%, 2=50-75%, 3=>75%) |  |  |  |  |  |
| Stricture (0=none, 1=single, can be passed, 2=Multiple, can be passed, 3=Cannot be passed) |  |  |  |  |  |
| SES CD | | | | |  |

**Supplemental Table 3. PROMIS Scale Global Health**

|  |  | **Excellent** | | **Very**  **good** | | | **Good** | | | **Fair** | | | **Poor** | | | |
| --- | --- | --- | --- | --- | --- | --- | --- | --- | --- | --- | --- | --- | --- | --- | --- | --- |
| **Global01** | **In general, would you say your health is:** | 5 | | | 4 | | | 3 | | | 2 | | | 1 | | |
| **Global02** | **In general, would you say your quality of life is:** | 5 | | | 4 | | | 3 | | | 2 | | | 1 | | |
| **Global03** | **In general, how would you rate your physical health?** | 5 | | | 4 | | | 3 | | | 2 | | | 1 | | |
| **Global04** | **In general, how would you rate your mental health, including your mood and your ability to think?** | 5 | | | 4 | | | 3 | | | 2 | | | 1 | | |
| **Global05** | **In general, how would you rate your satisfaction with your social activities and relationships?** | 5 | | | 4 | | | 3 | | | 2 | | | 1 | | |
| **Global09** | **In general, please rate how well you carry out your usual social activities and roles. (This includes activities at home, at work and in your community, and responsibilities as a parent, child, spouse, employee, friend, etc.)** | 5 | | | 4 | | | 3 | | | 2 | | | 1 | | |
|  | | **Completely** | | **Mostly** | | | **Moderately** | | | **A little** | | | **Not at all** | | | |
| **Global06** | **To what extent are you able to carry out your everyday physical activities such as walking, climbing stairs, carrying groceries, or moving a chair?** | 5 | | | 4 | | | 3 | | | 2 | | | 1 | | |
| **In the past 7 days…** | | **Never** | | | **Rarely** | | | **Sometimes** | | | **Often** | | | **Always** | | |
| **How often have you been bothered by emotional problems such as feeling anxious, depressed or irritable?** |  | 5 | | | 4 | | | 3 | | | 2 | | | 1 | | |
| **How would you rate your fatigue on average?** |  | 5 | | | 4 | | | 3 | | | 2 | | | 1 | | |
| **How would you rate your pain on average?** |  | 0  No pain | 1 | | 2 | 3 | | 4 | 5 | | 6 | 7 | | 8 | 9 | 10  Worst pain imaginable |

**Supplemental Table 4. PROMIS Distress-Anxiety**

| **Please respond to each question or statement by marking one box per row. In the past 7 days…** | | **Never** | **Rarely** | **Sometimes** | **Often** | **Always** |
| --- | --- | --- | --- | --- | --- | --- |
| **1** | **I felt fearful** |  |  |  |  |  |
| **2** | **I found it hard to focus on anything other than my anxiety** |  |  |  |  |  |
| **3** | **My worries overwhelmed me** |  |  |  |  |  |
| **4** | **I felt uneasy** |  |  |  |  |  |

**Supplemental Table 5. PROMIS Emotional Distress-Depression**

| **Please respond to each question or statement by marking one box per row. In the past 7 days...** | | **Never** | **Rarely** | **Sometimes** | **Often** | **Always** |
| --- | --- | --- | --- | --- | --- | --- |
| **1** | **I felt worthless** |  |  |  |  |  |
| **2** | **I felt helpless** |  |  |  |  |  |
| **3** | **I felt depressed** |  |  |  |  |  |
| **4** | **I felt hopeless** |  |  |  |  |  |

**Supplemental Table 6. PROMIS Fatigue**

| **Please respond to each question or statement by marking one box per row. During the past 7 days…** | | **Not at all** | **A little bit** | **Somewhat** | **Quite a bit** | **Very much** |
| --- | --- | --- | --- | --- | --- | --- |
| **1** | **I feel fatigued** |  |  |  |  |  |
| **2** | **I have trouble starting things because I am tired** |  |  |  |  |  |
| **In the past 7 days…** | | | | | | |
| **3** | **How run-down did you feel on average?** |  |  |  |  |  |
| **4** | **How fatigued were you on average?** |  |  |  |  |  |

**Supplemental Table 7. PROMISE Pain Interference**

| **Please respond to each question or statement by marking one box per row. In the past 7 days…** | | **Not at all** | **A little bit** | **Somewhat** | **Quite a bit** | **Very much** |
| --- | --- | --- | --- | --- | --- | --- |
| **1** | **How much did pain interfere with your day-to-day activities?** |  |  |  |  |  |
| **2** | **How much did pain interfere with work around the home?** |  |  |  |  |  |
| **3** | **How much did pain interfere with your ability to participate in social activities?** |  |  |  |  |  |
| **4** | **How much did pain interfere with your household chores?** |  |  |  |  |  |

**Supplemental Table 8. PROMISE Sleep Disturbance**

| **Please respond to each question or statement by marking one box per row. In the past 7 days…** | | **Very poor** | **Poor** | **Fair** | **Good** | **Very good** |
| --- | --- | --- | --- | --- | --- | --- |
| **1** | **My sleep quality was** |  |  |  |  |  |
| **In the past 7 days…** | | **Not at all** | **A little bit** | **Somewhat** | **Quite a bit** | **Very much** |
| **2** | **My sleep was refreshing** |  |  |  |  |  |
| **3** | **I had a problem with my sleep** |  |  |  |  |  |
| **4** | **I had difficulty falling asleep** |  |  |  |  |  |

**Supplemental Table 9. PROMISE Physical Function**

|  | **No difficulty** | **A little difficulty** | **Some difficulty** | **A lot of difficulty** | **Unable to do** |
| --- | --- | --- | --- | --- | --- |
| **Are you able to do chores such as vacuuming or yard work?** |  |  |  |  |  |
| **Are you able to go up and down the stairs at a normal pace?** |  |  |  |  |  |
| **Are you able to go for a walk for at least 15 minutes?** |  |  |  |  |  |
| **Are you able to run errands and shop?** |  |  |  |  |  |
| **Does your health now limit you in doing 2 hours of physical labor?** |  |  |  |  |  |
| **Does your health now limit you in doing moderate work around the house like vacuuming, sweeping floors or carrying in groceries?** |  |  |  |  |  |

**Supplemental Table 10. IBD Self-Efficacy**

| Over the past 2 weeks, how confident have you felt in your ability to perform each of the following tasks?  1 2 3 4 5 6 7 8 9 10  not sure at all somewhat sure totally sure |
| --- |
| Managing your stress and emotions |
| 1. Keep from getting stressed? |
| 2. Do something to make yourself less stressed? |
| 3. Keep from getting discouraged? |
| 4. Do something to make yourself feel better when discouraged? |
| 5. Keep from feeling sad or down in the dumps? |
| 6. Do something to make yourself feel better when sad? |
| 7. Keep sadness or anxiety from interfering? |
| 8. Do something to make yourself feel better when your sadness or anxiety interferes? |
| 9. Get emotional support from family or friends? |
| Managing your medical care |
| 10. Follow the instructions for your prescription medications? |
| 11. Take your prescription medication at the appropriate times? |
| 12. Take the medications to prevent a flare up of your IBD as directed? |
| 13. Work with your doctor or nurse to reach an agreement on a treatment plan? |
| 14. Ask your doctor about your illness? |
| 15. Discuss openly with your doctor any problems related to your medications? |
| 16. Work out differences with your doctor? |
| 17. Ask your doctor about your medications? |

**Supplemental Table 11. Healthcare utilization**

| 1. In the past 12 months, have you been a patient overnight in a hospital, nursing home or convalescent home for management of your Crohn’s disease, ulcerative colitis, or IBD? Yes/No/Don’t know 2. If yes, for how many nights in the past 12 months? 3. Not counting when you were hospitalized, in the past 12 months, have you been evaluated in an urgent care or emergency room for management of your Crohn’s disease, ulcerative colitis, or IBD? Yes/No/Don’t know 4. If yes, for how many days in the past 12 months? 5. In the past 12 months, have you undergone a bowel resection or creation of an ostomy (ileostomy, colostomy) for management of your Crohn’s disease, ulcerative colitis, or IBD? Yes/No/Don’t know 6. If yes, how many surgical procedures? 7. Not counting when you were an overnight patient, in the past 12 months, how many times have you seen or talked on the telephone about your Crohn’s disease, ulcerative colitis, or IBD with a family doctor or general practitioner? 8. Not counting when you were an overnight patient, in the past 12 months, how many times have you seen or talked on the telephone about your Crohn’s disease, ulcerative colitis, or IBD with a gastroenterologist or surgeon (Crohn’s, ulcerative colitis, IBD specialist)? 9. In the past 12 months, how many times have you had an endoscopy, sigmoidoscopy, or colonoscopy for evaluation of your Crohn’s disease, ulcerative colitis, or IBD? 10. In the past 12 months, how many times have you had a CT (CAT) scan or MRI for evaluation of your Crohn’s disease, ulcerative colitis, or IBD? 11. In the past 12 months, how many times have you had blood drawn or stool testing for evaluation of your Crohn’s disease, ulcerative colitis, or IBD? 12. In the past 12 months, have you been given a prescription of steroids (prednisone, methylprednisolone, budesonide) for treatment of your Crohn’s disease, ulcerative colitis, or IBD? Yes/No     1. If Yes, how many days in the last 12 months have you received steroids? |
| --- |
